# Deploying the Behavioral and Environmental Sensing and Intervention for Cancer Smart Health System to Support Patients and Family Caregivers in Managing Pain: Feasibility and Acceptability Study

**DOI:** 10.2196/36879

**Published:** 2022-08-09

**Authors:** Virginia LeBaron, Ridwan Alam, Rachel Bennett, Leslie Blackhall, Kate Gordon, James Hayes, Nutta Homdee, Randy Jones, Kathleen Lichti, Yudel Martinez, Sahar Mohammadi, Emmanuel Ogunjirin, Nyota Patel, John Lach

**Affiliations:** 1 University of Virginia School of Nursing Charlottesville, VA United States; 2 Massachusetts Institute of Technology Cambridge, MA United States; 3 University of Virginia School of Medicine Charlottesville, VA United States; 4 Virginia Commonwealth University Health Richmond, VA United States; 5 Trident Systems, Inc Fairfax, VA United States; 6 Faculty of Medical Technology Mahidol University Nakhon Pathom Thailand; 7 University of Virginia School of Engineering & Applied Science Charlottesville, VA United States; 8 Penn Medicine, University of Pennsylvania Health System Philadelphia, PA United States; 9 The George Washington University School of Engineering & Applied Science Washington, DC United States

**Keywords:** mobile health, mHealth, smart health, cancer, pain, palliative care, family caregiver, remote monitoring, feasibility and acceptability, rural

## Abstract

**Background:**

Distressing cancer pain remains a serious symptom management issue for patients and family caregivers, particularly within home settings. Technology can support home-based cancer symptom management but must consider the experience of patients and family caregivers, as well as the broader environmental context.

**Objective:**

This study aimed to test the feasibility and acceptability of a smart health sensing system—Behavioral and Environmental Sensing and Intervention for Cancer (BESI-C)—that was designed to support the monitoring and management of cancer pain in the home setting.

**Methods:**

Dyads of patients with cancer and their primary family caregivers were recruited from an outpatient palliative care clinic at an academic medical center. BESI-C was deployed in each dyad home for approximately 2 weeks. Data were collected via environmental sensors to assess the home context (eg, light and temperature); Bluetooth beacons to help localize dyad positions; and smart watches worn by both patients and caregivers, equipped with heart rate monitors, accelerometers, and a custom app to deliver ecological momentary assessments (EMAs). EMAs enabled dyads to record and characterize pain events from both their own and their partners’ perspectives. Sensor data streams were integrated to describe and explore the context of cancer pain events. Feasibility was assessed both technically and procedurally. Acceptability was assessed using postdeployment surveys and structured interviews with participants.

**Results:**

Overall, 5 deployments (n=10 participants; 5 patient and family caregiver dyads) were completed, and 283 unique pain events were recorded. Using our “BESI-C Performance Scoring Instrument,” the overall technical feasibility score for deployments was 86.4 out of 100. Procedural feasibility challenges included the rurality of dyads, smart watch battery life and EMA reliability, and the length of time required for deployment installation. Postdeployment acceptability Likert surveys (1=strongly disagree; 5=strongly agree) found that dyads disagreed that BESI-C was a burden (1.7 out of 5) or compromised their privacy (1.9 out of 5) and agreed that the system collected helpful information to better manage cancer pain (4.6 out of 5). Participants also expressed an interest in seeing their own individual data (4.4 out of 5) and strongly agreed that it is important that data collected by BESI-C are shared with their respective partners (4.8 out of 5) and health care providers (4.8 out of 5). Qualitative feedback from participants suggested that BESI-C positively improved patient-caregiver communication regarding pain management. Importantly, we demonstrated proof of concept that seriously ill patients with cancer and their caregivers will mark pain events in real time using a smart watch.

**Conclusions:**

It is feasible to deploy BESI-C, and dyads find the system acceptable. By leveraging human-centered design and the integration of heterogenous environmental, physiological, and behavioral data, the BESI-C system offers an innovative approach to monitor cancer pain, mitigate the escalation of pain and distress, and improve symptom management self-efficacy.

**International Registered Report Identifier (IRRID):**

RR2-10.2196/16178

## Introduction

### Background

Pain is a pervasive problem in advanced-stage cancer, occurring in almost 100% of patients [1] and undertreated in most (close to 70% of patients [2]). Complicating this reality is the fact that most cancer symptom management occurs in the home setting, often requiring significant support and help from family caregivers, who may be ill-prepared to take on this role [3,4]. The distress experienced by family caregivers in helping manage symptoms, especially difficult cancer pain, is well documented [5-10], as is the multitude of negative physical and emotional sequelae of poorly managed pain [11-13]. Ensuring equitable access to pain management requires innovative approaches that capitalize on low-burden home-based technologies that can support both patients and family caregivers. One critical lesson from the COVID-19 pandemic is the importance and great potential of remotely providing quality health care [14]. Sensing systems that can effectively monitor and prevent escalation of difficult symptoms at home, such as cancer pain, provide a powerful opportunity to reduce patient and caregiver distress, as well as unwanted emergency room visits and hospitalizations [15-23].

### Objectives

**Figure 1:**
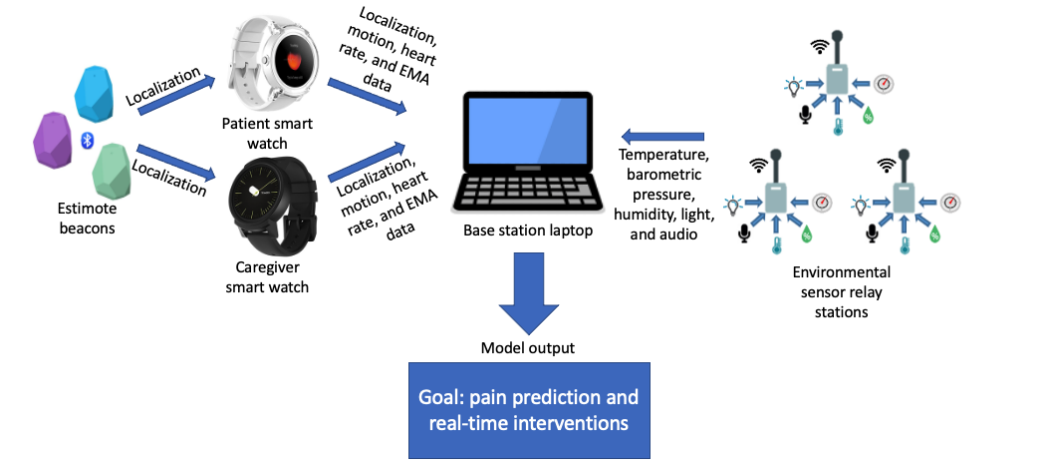
BESI-C system architecture used for feasibility and acceptability testing.

This study aimed to address the need for improved cancer pain management and represents a multiphase, interdisciplinary effort to design and test an in-home smart health remote monitoring system known as the Behavioral and Environmental Sensing and Intervention for Cancer (BESI-C). Our research has a particular focus on supporting the pain management needs of patients with advanced cancer and their family caregivers in rural settings, a population with well-documented disparities and challenges related to symptom management [24-27]. The overall research protocol [28] and user-centered design process [29] for BESI-C have been reported in detail elsewhere. Briefly, BESI-C is an end-to-end sensing system that consists of (1) physical components (smart watches, environmental sensors, and localization beacons) deployed in patient homes to gather physiological, behavioral, and contextual data regarding pain events from the perspective of both patients and family caregivers and (2) an approach for data analytics (Figure 1). The long-term clinical goal of BESI-C is to successfully predict pain episodes and deliver real-time tailored interventions to reduce distress and enhance self-efficacy in managing pain for both patients and caregivers, as well as sharing relevant data with stakeholders to inform personalized care management decisions. The broader aim of BESI-C is to reduce cancer health disparities by increasing equitable access to quality and compassionate cancer pain management. This manuscript presents the results of feasibility and acceptability testing of BESI-C and offers “lessons learned” for others engaged in similar digital health research.

## Methods

### Overall Study Design

This descriptive study assessed the feasibility and acceptability of the BESI-C system. Feasibility was operationalized *procedurally* as (1) logistic barriers related to in-home deployment (eg, structural constraints within the dyad home related to placing environmental sensors) and (2) participant recruitment and attrition rates and *technically* as (3) the fidelity of data capture, recorded as a composite score after each deployment. Acceptability was operationalized as dyad perceptions and receptivity to BESI-C and assessed at the time of removal of BESI-C from a dyad’s home by (1) a Likert-style survey and (2) structured interview questions asking about general experiences with the system.

### Ethics Approval

Ethics approval was granted by the University of Virginia Health Sciences Institutional Review Board (HSR IRB 21017), and all participants provided informed consent before data collection. The participants were shown prototypes or pictures of the BESI-C system during the informed consent process to better understand the project.

### Setting

Patients and family caregivers were recruited from an outpatient palliative care clinic at an academic medical center in the southeastern United States. BESI-C was deployed in patient and caregiver homes living in Central Virginia between April 2019 and December 2019 (before the COVID-19 pandemic).

### Sample

Our goal was to recruit patients and family caregivers coping with difficult cancer-related pain in a home setting. Therefore, we used a purposive sampling technique [30], and patient inclusion criteria included: (1) a diagnosis of locally advanced or metastatic malignancy, (2) currently taking prescribed opioid medications (eg, morphine type medications) for cancer-related pain, (3) scores of ≥6 on National Institutes of Health PROMIS (Patient-Reported Outcomes Measurement Information System) Cancer Pain Interference measures (a composite score assessed at each palliative care clinic visit to identify patients experiencing difficult pain) [31,32] or the standard 0 to 10 pain numeric rating scale, and (4) a primary informal (nonpaid; family, defined broadly) caregiver who helps manage their care and symptoms at home. Both patients and caregivers were aged ≥18 years, English speaking, and did not have cognitive or visual deficits or mental health issues that would preclude their ability to participate in the study. We excluded patients and caregivers who did not live in a private residence (eg, assisted living facility or nursing home), as we needed the ability to set up BESI-C without interfering with facility protocols or regulations. Palliative care clinicians helped screen and confirm the clinical eligibility of potential study participants.

### Data Collection Procedures

After patients and caregivers provided informed consent, basic clinical and demographic data were collected, and a time was scheduled to deploy BESI-C in their homes. A team consisting of clinicians (1 nurse faculty and 1 nursing student) and technicians (1-2 engineering students) traveled to participant homes to set up the BESI-C system and provide education regarding system use. The first author (VL) maintained a detailed audit log to record procedural and technical challenges related to each deployment.

Participants were asked to maintain and use the BESI-C system in their homes for 10 to 14 days. During deployment, remote system monitoring was performed by our technical team (using the software platform TeamViewer), and participants had a study phone number to call if they had problems or questions. All data streams were deidentified and labeled only by the study ID number. Our team also provided brief, periodic check-ins every 3 to 4 days via telephone calls or text (depending on dyad preference) or as needed, if technical issues arose. Both patients and caregivers were asked to keep a ground truth daily log during deployment to record key events that may influence pain or functionality of the system (eg, prolonged power outage, hospital admission, or injury or fall).

During deployment, streaming data were passively collected from the smart watches worn by both patients and caregivers (heart rate and motion), environmental sensors (ambient noise, humidity, barometric pressure, light, and temperature data), and Bluetooth beacons (to help localize dyad positions within the home and in relation to each other). Active data (ie, requiring user engagement) were collected from ecological momentary assessments (EMAs) delivered via smart watches, including on-demand EMAs that allowed patients and caregivers to record and describe patient pain events from their own perspective when they occurred, as well as 30-minute follow-up pain reassessment EMAs (7 items). The smart watches also generated a daily scheduled EMA survey (12 items) to assess other factors over the past 24 hours that can influence pain, such as self-reported sleep quality and mood. EMAs were purposely designed to be fast and easy to complete and used simple Likert scale (0-10) or categorical response options (eg, “not at all,” “a little,” “fairly,” or “very”). Details of EMA data collection are the focus of a subsequent publication.

At the conclusion of the deployment, our team returned to the participants’ home, removed the equipment, and assessed the patient and caregiver experience with BESI-C by a structured interview and a Likert-style survey administered to both the patient and the family caregiver. Responses were captured verbally and recorded by pen-and-paper by study team members for deployments 1 to 4 and via an iPad (Apple Inc) for deployment 5. All participants were asked 13 Likert-style survey questions designed to assess their opinions regarding perceptions of system helpfulness (n=1), burden and privacy concerns (n=3), data sharing preferences (n=3), ease of using smart watches to mark and describe pain events (n=3), concerns regarding environmental sensors (n=1), and perceived impact of the system on cancer pain management and communication with their partner (n=2). Optional free-text responses within the survey allowed participants to expand on their answers or provide suggestions regarding system components. Structured interview questions (added after deployment 1, as we realized that more context was needed for some of the Likert scale survey items) provided additional opportunities for participants to discuss their experiences with the system. As the goal of this study was to understand the feasibility and acceptability of very specific features of our system architecture to guide future work, we opted to create a customized survey and interview guide [33], informed conceptually by other mobile health and technology evaluation tools, such as the System Usability Scale [34] and Mobile App Rating Scale [35]. The dyads received a US $50 gift card as compensation for their time.

### Data Analysis Procedures

#### Survey and Interview Data

Postdeployment survey and structured interview data collected from patients and caregivers were verified and entered into Qualtrics for data management and storage. Quantitative responses were exported to SPSS (version 26.0; IBM Corporation), and basic descriptive statistics were run, including frequency counts and percentages for demographic data and individual and category means for Likert scale items. Independent sample *t* tests (2-tailed) were performed across all individual and category variables to assess statistically significant differences (Cronbach α=.05) between patient and caregiver mean scores. Likert scale survey items in which the respondent selected the option “don’t know” were omitted from analysis. Textual data (open-text survey and structured interview responses) were exported into Microsoft Word and organized into clusters using a basic descriptive content analysis approach that mapped to the questions asked (eg, all responses to a particular question were grouped together and reviewed for patterns). Our goal with the analysis of open-ended responses was not to conduct a qualitative analysis with a high level of abstraction, but instead, consistent with a descriptive approach, to stay close to our data and concretely understand participant responses [36].

#### Calculating Data Fidelity

**Figure 2:**
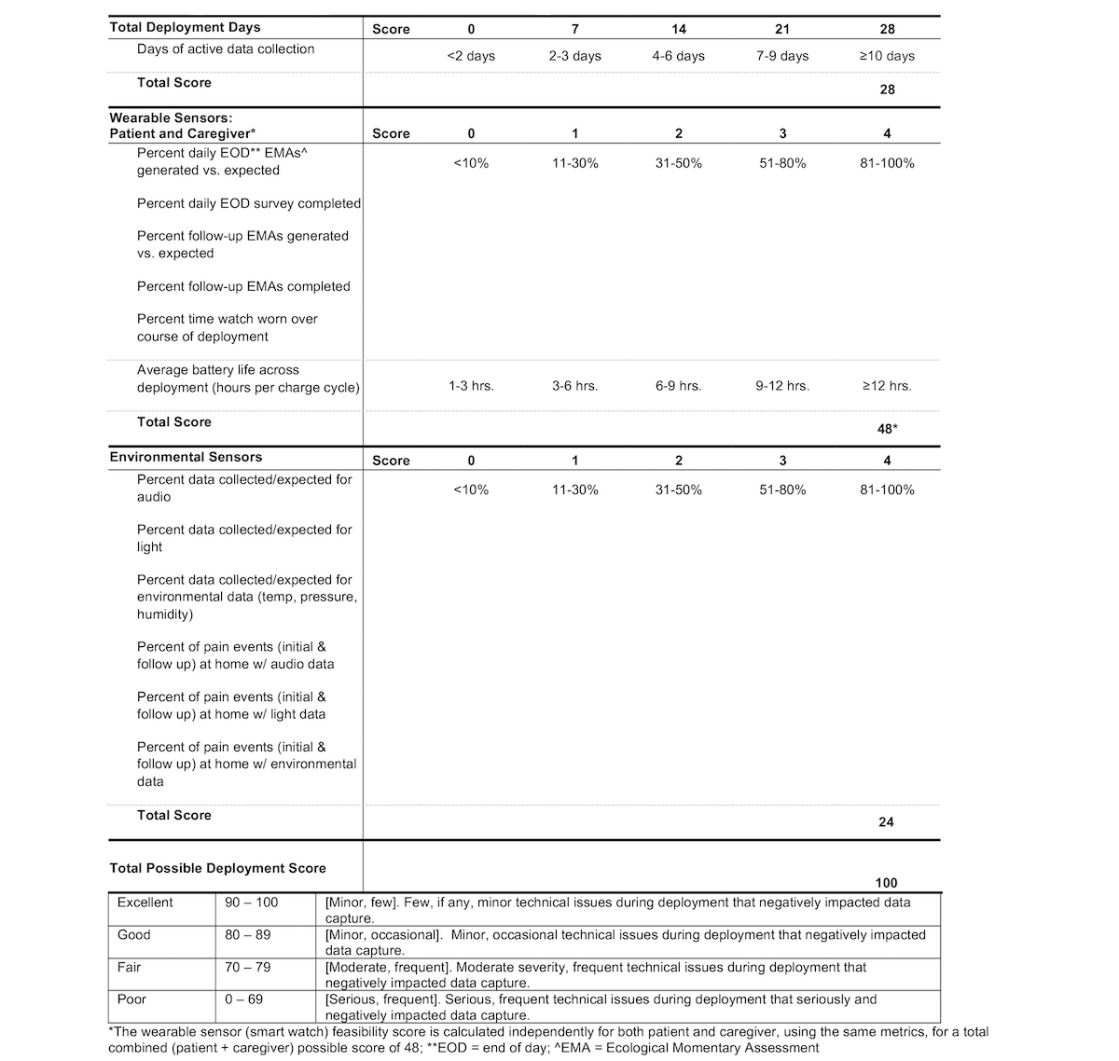
Template for the “BESI-C Performance Scoring Instrument” to assess technical feasibility of the system. EMA: ecological momentary assessment; EOD: end of day.

We created a BESI-C Performance Scoring Instrument (Figure 2) to quantify the fidelity of data capture for each deployment. Conceptually, this tool was inspired by symptom assessment tools commonly used in clinical practice to better understand the health and functioning of individuals, such as the Memorial Symptom Assessment Scale [37] or the Eastern Cooperative Oncology Group (ECOG) Performance Status Scale [38]. Relatedly, the goal of our BESI-C Scoring Instrument was to understand the “health and functioning” of the BESI-C system. The BESI-C Performance Scoring Instrument is organized by the key components of the system architecture with corresponding feasibility parameters established by team consensus for poor or missing (score of 0), fair (score of 1), average (score of 2), good (score of 3), or excellent (score of 4) outcomes, with the highest possible score of 100. The following four categories were captured: (1) days of active data collection, (2) EMA reliability and data input from the patient’s smart watch, (3) EMA reliability and data input from the caregiver’s smart watch, and (4) reliability and data input from environmental sensors. The “total deployment days” category, which included 1 key metric, was weighted appropriately to ensure it was equally considered along with other category feasibility metrics. Our goal was to collect data between 10 and 14 days for each deployment. Specific details and examples of how each metric was calculated are included in Multimedia Appendix 1.

## Results

### Sample Characteristics

A total of 10 individuals (5 dyads of patients and their primary family caregivers) completed BESI-C feasibility and acceptability deployments (Table 1). Overall, most participants were aged between 55 and 74 years (8/10, 80%), female (6/10, 60%), and living in a rural setting (8/10, 80%). A total of 60% (6/10) of the participants identified as White; 40% (4/10) identified as Black or African American. All caregivers, except 1, were female (4/5, 80%), and all were spouses of the patients (5/5, 100%). A total of 3 out of 5 (60%) patients were diagnosed with head and neck cancer, whereas the others included colorectal (1/5, 20%) and lung (1/5, 20%) cancers. The average baseline numeric patient pain score [39] was 6.8 out of 10. A total of 3 out of 5 (3/5, 60%) patients self-reported their ECOG performance score [38] as 1, “symptomatic and ambulatory”; one patient (1/5, 20%) self-reported an ECOG score of 2, “ambulatory 50% of the time, some help needed”; one patient (1/5, 20%) did not self-report an ECOG score.

**Table 1:** Demographic characteristics of patient and caregiver sample.

Demographic variable		Total (N=10), n (%)	Patients (n=5), n (%)	Caregivers (n=5), n (%)
**Age band (years)**				
	45-54	1 (10)	1 (20)	0 (0)
	55-64	4 (40)	2 (40)	2 (40)
	65-74	4 (40)	2 (40)	2 (40)
	75-84	1 (10)	0 (0)	1 (20)
Rural^a^		8 (80)	4 (80)	4 (80)
**Sex**				
	Female	6 (60)	2 (40)	4 (80)
	Male	4 (40)	3 (60)	1 (20)
**Race**				
	Black or African American	4 (40)	2 (40)	2 (40)
	White	6 (60)	3 (60)	3 (60)
**Ethnicity**				
	Latino or Hispanic	0 (0)	0 (0)	0 (0)
	Non-Latino or non-Hispanic	10 (100)	5 (100)	5 (100)
**Highest education level**				
	Less than high school	1 (10)	1 (20)	0 (0)
	High school graduate	2 (20)	0 (0)	2 (40)
	Some college	5 (50)	3 (60)	2 (40)
	Professional or graduate degree	2 (20)	1 (20)	1 (20)
**Current employment**				
	Full-time	3 (30)	2 (40)	1 (20)
	Retired	6 (60)	2 (40)	4 (80)
	Other	1 (10)	1 (20)	0 (0)
Relationship with patient: spouse		N/A^b^	N/A	5 (100)
**Primary cancer diagnosis**				
	Head and neck	N/A	3 (60)	N/A
	Colorectal	N/A	1 (20)	N/A
	Lung	N/A	1 (20)	N/A

^a^Rural as identified by Centers for Medicaid and Medicare Services; Rural Health Information Hub [40].

^b^N/A: not applicable.

### Feasibility

#### Logistical and Technical Deployment Challenges

Logistic deployment barriers included the rural location of dyads, which involved challenges coordinating time-intensive trips to dyad homes along with internet stability issues and the length of time it took to set up the system, which varied according to the size of the home and other unanticipated factors. For example, in some homes, limited or poorly situated electrical outlets to plug in environmental sensors created challenges and added time to system installation. Table 2 summarizes the key logistic and technical barriers that occurred at the time of installation, during deployment, and at the time of system removal or teardown, along with subsequent iterative system changes or improvements.

**Table 2:** Summary of key technical and procedural deployment challenges and resulting iterative changes.

Deployment number	Total days of active data collection	Technical and procedural deployment challenges	How system and deployment procedures were changed or improved and lessons learned
1. Install: 190 min; teardown: 35 min	12	Lengthy installation time (due in part to smart watches not properly paired with base station; teaching took 45 min). Unable to remotely monitor smart watches due to bug in code logic; this required 2 members of engineering team to make additional trip to dyad home to fix. Inconsistent delivery of EMAs^a^ on caregiver smart watch. Patient stopped wearing smart watch in final days of deployment due to a fall.	Created standardized predeployment protocol checklists for both engineers and nurses to streamline deployment installation (eg, asking better dyad screening questions about size of home; developed environmental sensor placement protocol). Cross-trained nurse team members to help engineers place environmental sensors to expedite installation process. Established time goal of 1 h for installation; 30 min for teardown. Revised structure of daily EMAs; decreased smart watch touchscreen sensitivity; added a “do not disturb/sleep” option on smart watch app. Created a “ground truth” daily log for patients and caregivers to record important events that may occur during deployment (such as a fall or injury).
2. Install: 75 min; teardown: 38 min	9	Patient reported they had stable internet, but this was not the case when we arrived in home. Mobile hot spot was set up. Smart watch battery life lasting 6-7 h (vs desired 10-12 h); patient smart watch had to be factory reset due to running out of power, which resulted in loss of data. EMAs not generating or coming at wrong time; smart watches not displaying correct date or time; base station went offline and did not connect properly to hot spot. Smart watches “locking” after deployment resulting in difficulty offloading collected data. Patient consented to study alone in clinic; caregiver unaware of pending deployment until study team arrived at dyad home.	Ask more detailed questions about internet and cellular service before in-home visit; be prepared to set up mobile hot spot if needed. Allow more time during installation for participants to practice using app and answering EMAs. Investigation regarding battery life undertaken. Avoid plugging in base station to switch-controlled electrical outlet. Implemented automatic data download script to download smart watch data when they are charging to prevent any data loss. Enhanced predeployment testing. Changed recruitment and consenting processes to ensure caregiver aware of scheduled deployment.
3. Install: 95 min; teardown: 45 min	12	Environmental sensors would not stick to wood paneling with standard 3M strips. Participant confusion regarding EMAs; did not feel like they could answer some questions properly. Issues with button press activation of EMAs due to patient neuropathy (numbness in fingers). Battery life of smart watches still problematic, lasting 4-5 h. Smart watches displaying correct data/time, but daily EMAs behaving inconsistently, not coming at all or generating at wrong time.	Ensure other measures are available to adhere environmental sensors to walls, such as sticky putty. Added “unsure” option to relevant EMAs. Changed all EMAs to "touch to wake" or screen tap. Refined sampling times for heart rate and accelerometer and operating system settings to optimize battery life. Changed daily EMA to be manually available between 5 PM to midnight with a reminder sent at 8:30 PM.
4. Install: 75 min; teardown: 47 min	14	Continued issues with daily EMAs not generating at correct times and smart watch battery life. Caregiver did not understand she should continue to wear the smart watch even if she is not physically with patient. Smart watch time going out of sync after battery dies. Safety concerns for study team related to unsecured firearms in dyad home.	System lock turned on to help with time sync issues with smart watches; code changed to help with processing power and accelerometer efficiency. Smart watch wearing instructions revised. Began deploying an Android smart phone to help sync the time and date on the smart watch when the smart watch battery dies. Created home-safety protocol for team.
5. Install: 100 min; teardown: 100 min^b^	15	Blue light on environmental sensor in bedroom kept patient awake at night. One Bluetooth beacon that was placed on top of refrigerator fell into the freezer. One environmental sensor lost connectivity to the system and was not able to be put back online. Patient smart watch not seen with remote monitoring; possibly due to system lock out turned off (to help with time sync issue and prevent smart watch from powering down) or from bug in code; follow-up EMAs not consistently being generated; random buzzes; long lag time with “touch-to-wake” feature of smart watch. New operating system update of the smart watches came with battery consumption reduction mode called “doze mode”; this interfered with EMAs being generated.	Ensure tape is placed over environmental sensors to prevent sleep disturbance. Caution with placement of Bluetooth beacons. Adding redundant environmental sensors in monitored rooms to ensure adequate data capture. Code changed to ensure smart watches do not go into “doze mode” and to address other inconsistencies with EMA delivery.

^a^EMA: ecological momentary assessment.

^b^Increased teardown time primarily due to particularly social or talkative dyad; also, iPads for survey data collection took longer to use with this deployment.

#### Fidelity of Data Capture

Table 3 summarizes the composite BESI-C performance scores for all the 5 deployments. Full deployment BESI-C Scoring Instruments for all 5 deployments are included in Multimedia Appendix 1. The overall performance deployment score across all categories and for all 5 deployments was 86.4 out of 100. The first deployment had the lowest overall total score (77 out of 100), with improvements in total performance scores for later deployments (89 out of 100, 89 out of 100, 89 out of 100, and 88 out of 100, respectively). The environmental or room sensors had the most consistent performance (24 out of 24 for each deployment). One deployment did not achieve a full score for the number of days of active data collection (deployment 2, score of 21 out of 28). Performance variability was greatest with smart watches, with a caregiver smart watch average score across all deployments of 16.4 out of 24, and a patient smart watch average score across all deployments of 19.4 out of 24.

**Table 3:** Behavioral and Environmental Sensing and Intervention for Cancer Performance Scoring Instrument composite scores for pilot deployments.

Category	Deployment 1, score	Deployment 2, score	Deployment 3, score	Deployment 4, score	Deployment 5, score	Category average, score
Total deployment days	28/28	21/28	28/28	28/28	28/28	26.6/28
Smart watch: patient	15/24	21/24	20/24	19/24	19/24	19.4/24
Smart watch: caregiver	10/24	20/24	17/24	18/24	17/24	16.4/24
Environmental or room sensors	24/24	24/24	24/24	24/24	24/24	24/24
Total deployment score	77/100	89/100	89/100	89/100	88/100	86.4/100

#### Participant Recruitment and Attrition

Participant recruitment was significantly disrupted by the COVID-19 pandemic (which required the complete cessation of recruitment after our fifth deployment; we had planned for 15). Screening for eligibility was complicated by inherent limitations within the electronic health record, which made it difficult to verify key eligibility criteria such as caregiver status. A total of 2 dyads signed consent but withdrew before deployment; one due to being too busy; the other dyad was lost to follow-up and unable to be contacted. In all, 80% (4/5) of dyads who signed the consent form and had the system installed completed the minimum (10 days) target length of data collection. One dyad (1/5, 20%) only completed 9 days of data collection, but this was due to technical failures that truncated data input versus voluntary attrition.

### Acceptability

#### Postdeployment Assessments: Quantitative

**Figure 3:**
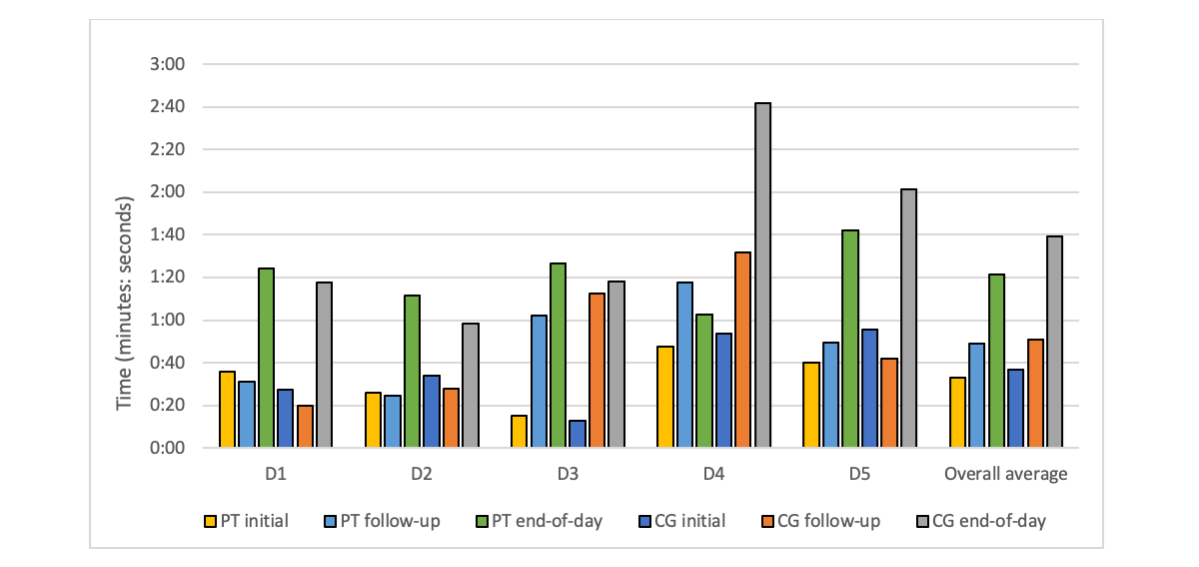
Average ecological momentary assessment (EMA) completion times per deployment and overall. EMAs recorded as taking >5 minutes to complete (n=28) were considered incomplete EMAs or outliers and were omitted from analysis. "PT initial" and "CG initial" refer to the first pain event the EMA recorded. "PT follow-up" and "CG follow-up" refer to the 30-minute pain reassessment EMA. "PT end of day" and "CG end of day" refer to the end-of-day summary survey EMA. CG: caregiver; D: deployment; PT: patient.

Postdeployment Likert surveys demonstrated that, overall, patients and caregivers perceived the BESI-C system to be helpful and low burden (Table 4). Specifically, on a scale of 1 (strongly disagree) to 5 (strongly agree), dyads agreed that BESI-C collected helpful data to better manage cancer pain (4.6 out of 5) and that it was easy to answer EMAs on the smart watch (4.3 out of 5) and remember to mark pain events in real time (4.4 out of 5) and expressed a willingness to answer more EMAs on the smart watch (4 out of 5). Completion times for initial and follow-up pain EMAs across all deployments were generally <1 minute (Figure 3), with slightly longer completion times for the daily end-of-day EMA, which was expected because this EMA survey included more questions. Overall, 283 unique initial pain events were reported, along with 106 follow-up pain reassessment EMAs. A total of 63 daily surveys were completed (Table 5). Further details of EMA results are the focus of a subsequent publication. Dyads disagreed that the system was a burden to themselves (1.5 out of 5) or their partner (1.7 out of 5) or violated their privacy (1.9 out of 5). Overall, dyads expressed a strong interest in data sharing (4.7 out of 5), with patients and caregivers equally agreeing about their desire to see their own data (4.4 out of 5), and even more strongly agreeing on the importance of sharing data with their respective partners (4.8 out of 5) and health care providers (4.8 out of 5). Interestingly, caregivers disagreed more strongly about the unobtrusiveness of the environmental sensors (3.4 out of 5) than patients (4.8 out of 5). Dyads disagreed that the BESI-C changed pain medication use (overall and patients: 2.2 out of 5; caregivers: 2.3 out of 5). Caregivers (4.4 out of 5) agreed more strongly than patients (2.6 out of 5) that recording pain events increased their awareness of pain. No statistically significant differences were found between the patient and caregiver responses (Cronbach α=.05).

Within Table 4, missing values are due to the patient or caregiver selected the response “do not know” or declined to answer (one patient, 1/5, 20% did not self-report an ECOG score).

**Table 4:** Comparison of postdeployment Likert survey mean scores by overall sample, patients, and caregivers (1=strongly disagree; 5=strongly agree).

Question asked of participant			Total (N=10)		Patients (n=5)		Caregivers (n=5)
Overall perceptions: I think BESI-C^a^ can collect helpful information to better manage cancer pain, mean (SD); n			4.6 (0.52); 10		4.6 (0.55); 5		4.6 (0.55); 5
**System burden, category mean (SD)**			1.7 (0.51)		1.7 (0.43)		1.7 (0.43)
	BESI-C system was a burden for me, mean (SD); n	1.5 (0.71); 10		1.4 (0.55); 5		1.6 (0.89); 5	
	BESI-C system was a burden for my partner, mean (SD); n	1.7 (0.71); 9		1.8 (0.50); 4		1.6 (0.89); 5	
	BESI-C system made me concerned about privacy, mean (SD); n	1.9 (0.99); 10		2 (0.71); 5		1.8 (1.30); 5	
**Data sharing preferences, category mean (SD)**			4.7 (0.44)		4.7 (0.47)		4.7 (0.47)
	I want to see the information collected by BESI-C about my experience, mean (SD); n	4.4 (0.84); 10		4.4 (0.89); 5		4.4 (0.89); 5	
	I think it is important to share information collected by BESI-C with my partner, mean (SD); n	4.8 (0.42); 10		4.8 (0.45); 5		4.8 0.45); 5	
	I think it is important to share information collected by BESI-C with health care providers, mean (SD); n	4.8 (0.44); 9		4.8 (0.45); 5		4.8 (0.50); 4	
Environmental sensors (I mostly forgot about the room sensors after the first day), mean (SD); n			4.1 (0.99); 10		4.8 (0.45); 5		3.4 (0.89); 5
**Smart watch or EMAs^b^, category mean (SD)**			4.2 (0.57)		4.5 (0.38)		4.0 (0.67)
	It was easy to answer questions on the smart watch, mean (SD); n	4.3 (0.82); 10		4.6 (0.55); 5		4.0 (1.00); 5	
	Remembering to mark pain events in the moment was easy, mean (SD); n	4.4 (0.70); 10		4.6 (0.55); 5		4.2 (0.84); 5	
	I would be willing to answer more questions on the smart watch, mean (SD); n	4 (0.94); 10		4.2 (0.84); 5		3.8 (1.10); 5	
**Pain, category mean (SD)**			2.9 (1.04)		2.4 (1.04)		3.4 (0.71)
	BESI-C changed the way I or the patient normally takes their pain medication, mean (SD); n	2.2 (1.48); 9		2.2 (1.64); 5		2.3 (1.50); 4	
	Recording pain events made me more aware of the pain I or the patient was feeling, mean (SD); n	3.5 (1.51); 10		2.6 (1.52); 5		4.4 (0.89); 5	

^a^BESI-C: Behavioral and Environmental Sensing and Intervention for Cancer.

^b^EMA: ecological momentary assessment.

**Table 5:** Total number of completed ecological momentary assessments (EMAs) per deployment by patient and caregiver.

	Deployment 1		Deployment 2			Deployment 3			Deployment 4			Deployment 5			Total, N
	Pt^a^	CG^b^	Pt	CG	Pt		CG	Pt		CG	Pt		CG		
Initial pain EMA	49	15	42	18	24		15	30		21	53		16	283	
Follow-up pain EMA^c^	25	5	15	7	19		9	15		3	5		3	106	
End-of-day EMA	5	4	6	4	10		6	6		3	12		7	63	
Total	79	24	63	29	53		30	51		28	70		26	452	

^a^Pt: patient.

^b^CG: caregiver.

^c^Pain reassessment EMAs generated 30 minutes after an initial pain EMA, if participant reported that the patient took pain medication.

#### Postdeployment Assessments: Qualitative

Write-in or free-text survey items revealed that participants found the BESI-C system beneficial, particularly in relation to dyadic communication. Questions inquiring about specific hardware components of the system architecture (smart watches, environmental sensors, base stations or laptops, and localization beacons) yielded minimal or no comments or suggestions. Most of the feedback from the participants involved the smart watch interface and its functionality. A caregiver expressed concern regarding how environmental sensors may be perceived by visitors to the home (“is the government spying on us?”) Both caregivers and patients acknowledged some frustration with the technical challenges with the smart watches, including battery life, occasional lag in the touch-to-wake screen tap feature, and inconsistency with EMA delivery. A caregiver expressed the desire for greater flexibility in describing unusual events that may influence pain.

Structured interviews allowed participants to more fully contextualize or expand upon their survey responses, and dyads largely reiterated perceptions documented in the free-text survey items, particularly related to technical inconsistencies with smart watch functioning. Despite technical glitches with the smart watch app interface, when asked, “about what percentage of the time did you wear the smart watch in a 24-hr period?” 40% (4/10) of the participants said 100% of the time, 30% (3/10) said 75% of the time, and 10% (1/10) said 85% of the time; this question was added after deployment 3, and so only answered by participants of deployments 4 and 5. A participant was particularly averse to wearing the smart watch as he explained, “he doesn’t wear a watch in general” and expressed a dislike for jewelry. Participants also expressed a desire for clearer instructions about wearing the smart watch and an interest in having the smart watch capture distress from symptoms other than pain, such as nausea. Because of the high degree of similarity in responses to free-text survey items and structured interview questions, qualitative feedback was integrated and is summarized in Table 6.

**Table 6:** Summary of postdeployment qualitative responses related to Behavioral and Environmental Sensing and Intervention for Cancer (BESI-C).

Question	Pt^a^	CG^b^
What was your general or overall impression of having BESI-C in your home?	“Just need to work out watch problems.” [Pt 1] “It was a painless event. Didn’t know it was there. Did like the way it followed up [about the pain] with the follow up-EMA.” [Pt 2] “The technical aspect was frustrating and inconsistent. Hard rating the pain since I was trying to stay ahead of the pain.” [Pt 3] “Didn’t bother us a bit.” [Pt 4] “Positive. Did not pay any attention to the equipment at all...This will be a great asset to patient dealing with pain. It makes you more aware of how important it is to manage pain properly and on a timely basis...” [Pt 5]	“Just needs to work more consistently.” [CG 1] “[Privacy concerns] got better over time... we adjusted.” [CG 2] “An interesting study and easy to use”; “equipment was inconsistent” (caregiver notes that they did not wear the smart watch to sleep). [CG 3] “Some days would work well, sometimes not. It’s not obvious when she’s in pain. When she was taking a pill I would guess she’d be in pain.” [CG 3] “Battery life [was an issue].” [CG 4] “Didn’t even know [environmental] sensors were here.” [CG 4] “I think it can help a lot of people out there who cannot get to a doctor when they’re really hurting and sick. Think you have a great invention here!” [CG 5]
What did you like about having BESI-C in your home? What did you dislike about having BESI-C in your home?	“Made me pay attention to what I was feeling and if my caregiver felt it.” [Pt 3] “Helped me communicate with [my partner] more; Felt like I was able to tell [my partner] I was in pain, not hiding it and not waiting to take pain medication.” [Pt 5] “Disliked watch. I don’t like wearing jewelry. Don’t wear a watch in general.” [Pt 4] “Lag time in watch turning on was frustrating. Watch went back to black screen before you could answer.” [Pt 5]	“It was easy, took little time out of the day.” [CG 3] “If it can help someone, I’m glad to do it.” [CG 4] “The watch didn’t bother me. [But] I had to remember to wear the watch. It wasn’t clear if I had to wear it if I wasn’t with [patient].” [CG 4]
What could be changed to make the BESI-C system better?	“Accuracy with watch date/time; end of day surveys.” [Pt 2] “Work to improve watch lag time.” [Pt 5]	“Longer charge on watch.” [CG 2] “Include nausea. [Pt] was having nausea and I was distressed but that wasn’t because she was in pain.” [CG 3] “Clearer instructions when to wear watches. When we were apart, wasn’t sure how to answer the questions.” [CG 4]
Did having BESI-C in your home impact or change how you communicated or interacted with your partner about pain? If so, how?	“We discussed pain more.” [Pt 2] “She asked more specific questions about my pain.” [Pt 4] “The system helped me take my medication on a more consistent basis before the pain built up to an intolerable level...`Before the BESI-C system I wouldn’t always communicate my pain with my caregiver in trying to prevent him from worrying. The system made me aware by not communicating I was doing the [opposite].” [Pt 5]	“I was paying more attention to the small things—like does she go sit down and rest? Raised awareness on pain management and how she looks and acts.” [CG 3] “This is a good way to communicate...It made her [patient] more aware to take the pain medication at the right time so the pain did not build up and get worse and she could tolerate it better.” [CG 5]
You had the BESI-C system in your home for (10-14) days. Would you be willing to have BESI-C in your home for longer? Why or why not?	Yes “The feeling of being monitored may be of benefit to me or others.” [Pt 2] “I want the equipment to be tweaked. I want to be able to explain things under unusual event. BESI-C makes sense to me, helps piece things together.” [Pt 3] No “It was enough time. Found [ground truth] log annoying. Should be less repetitious—just note what has changed or unusual. Not so many reminders on watch.” [Pt 5]	Yes “Sure. It was easy, didn’t take much time. Interesting in the beginning. Wanted to help in research. I liked the ‘level of distress’ question.” [CG 3] “If it’s helping us or others, then yes.” [CG 4] No “People were asking about what the sensors were for, asking us ‘is the government watching us?’” [CG 2]

^a^Pt: patient.

^b^CG: caregiver.

## Discussion

### Summary of Findings and Potential Impact

In this study, we demonstrated the acceptability and feasibility of deploying a smart health system, BESI-C, in the homes of adults with advanced cancer, to collect holistic and heterogenous sensing data from patients, caregivers, and the home environment. Importantly, our findings suggest an innovative approach to supporting home-based symptom self-management for cancer pain, promoting patient and caregiver self-efficacy, and strengthening the relationship between caregivers and care recipients—all critical and persistent gaps in oncology care [41,42]. More specifically, our research contributes to advancing the science of remote oncology care [43-47] and extends current efforts to leverage technology to monitor and manage cancer pain [48-51] by providing data that can inform future interventions. For example, by monitoring environmental and contextual factors in the home that may influence pain, BESI-C could prompt a patient or caregiver to implement a low-burden, high-impact environmental modification to reduce pain, such as adjusting the room temperature. In addition, BESI-C concurrently incorporates the perspective of *both* the patient and the family caregiver via smart watches programmed with a custom app to collect participant-reported EMA data, as well as passive physiological data. This is critically important, as a holistic understanding of the family caregiver experience in the context of the patient experience is essential for designing effective cancer interventions [3,52]. Integrating data from BESI-C to develop a comprehensive understanding of cancer pain experience at home facilitates the design of multidimensional interventions that can be tailored to the patient, caregiver, dyad, or home itself. The BESI-C approach offers unique benefits for rural populations who may live far from cancer care centers and may reduce disparities related to access to quality cancer pain care. In addition, the BESI-C system can provide critical support to clinicians by providing holistic, longitudinal data related to the pain experience at home (versus relying on a cross-section of recollection by patients or caregivers when they present for an outpatient clinic visit). Below, we discuss the implications of our findings and specific lessons learned related to acceptability and feasibility.

### Acceptability

We found that patients and caregivers coping with serious, advanced cancer *will* mark pain events in real time using a smart watch and that they find this activity meaningful and not overly burdensome. This is a noteworthy finding given the severity of illness experienced by palliative care patient populations, which can make data collection extremely difficult or impossible [53-55]. We believe this underscores and confirms the value patients and caregivers place on meaningful self-reported outcomes [44,56,57] and validates other work seeking to use EMAs to collect data about cancer pain [48]. We also believe that participants’ acceptance of answering EMAs about pain in real time was enhanced by our intentional choice to use smart watches versus a mobile phone app. Although mobile smartphones are ubiquitous, we wanted an even more direct and straightforward way (ie, a device “attached” to the person) for participants to record difficult symptoms in real time; our results confirm that the smart watch is an effective method for this type of symptom data collection. We did have a patient who was uniquely averse to wearing a smart watch, and future iterations of the system architecture could potentially offer a smartphone mobile app option for such patients. Our work in this area makes important contributions related to the use of smart watches for remote health monitoring by collecting both continuous physiological data as well as EMA data from actual patients with cancer [58-60].

We also learned that once participants became accustomed to the smart watch interface (which usually took only a couple of practice rounds), they were able to answer the EMAs very quickly, generally in <30 seconds. Postdeployment assessments also revealed that the participants were willing to answer additional EMAs. This was helpful information, as we purposely designed the EMAs for this study to be as streamlined and brief as possible to enhance adherence and reduce participant burden; this required making difficult choices about questions to include and ones to omit. Confirmation that we had latitude to add questions increased our confidence to add EMAs to the next iteration of our smart watch app, such as important questions about the use of nonpharmacological measures taken to reduce pain and other co-occurring symptoms, such as fatigue. Importantly, we also confirmed that patients and caregivers not only want to share collected data with their health care providers but that they wish to see their own data and for their partners to see their data. This is an important finding, as prior work has demonstrated challenges in ensuring health care providers understand and act upon patient-reported outcome data [61]. Given this reality, we concur with Villegas et al [48] and suggest that a more effective (or at least equally important) strategy is to focus on how remote monitoring data can inform real-time intervention strategies delivered directly to patients and caregivers for more empowered symptom self-management. We hypothesize that different “buckets” of data exist, and who needs access to these data—when, and how, and in what ways—will vary, temporally and by end user. For example, there are likely data most relevant to the patient themselves, data best mutually shared between patients and family caregivers, data helpful for the caregiver only, data best shared between health care providers and family caregivers, and data most helpful to health care providers. A key element of future work will be to explore more robustly how, when, and to whom to present relevant data visualizations and how they can best inform interventions.

Another interesting finding is that BESI-C may influence dyadic communication related to cancer pain management and medication use. Unfortunately, we were unable to interpret the direction of these Likert scale survey items (eg, caregivers, 4.4 out of 5, agreed more strongly than patients, 2.6 out of 5, that “recording pain events increased awareness of pain”—but whether this was considered positive or negative by the participant is unclear; these items have since been revised for future work). Qualitative responses, however, were able to shed light on these ambiguous quantitative results. In the postdeployment interviews, both patients and caregivers discussed that BESI-C made them more attuned to their partner’s experience and created more awareness of pain in a way that facilitated earlier, more proactive symptom management and enhanced communication. Navigating challenging cancer symptoms is an immensely stressful experience for patients and caregivers, and the potential for BESI-C to lessen distress by improving interpersonal communication is exciting.

Importantly, we also learned to provide clearer instructions regarding marking pain events on smart watches. With our first deployments, we purposely did not provide overly specific instructions regarding how and when participants should mark pain events. This created confusion for some participants, who were unsure when exactly they were supposed to mark pain events and what exactly constituted a “cancer-related pain event,” particularly if the patient experienced some level of constant, baseline pain (which is normative for many patients with cancer). In response to this, we became clearer that our on-demand EMAs were best designed to capture “breakthrough pain”—pain that increases or “breaks through” a patient’s baseline level of pain, which is notoriously difficult to assess and manage owing to its short duration, intensity, and unpredictable nature [18,48,62,63]. Once we had a better understanding of this, we revised our instructions to participants and explained, “Tap the screen on the smart watch to report an episode of cancer pain. You can consider a pain event as one in which the pain has increased from what it was previously and that you feel requires attention. Mark the pain event as close to when it occurs as possible. You do not need to report pain clearly unrelated to cancer (eg, stubbing a toe)*.*” Recognizing the BESI-C’s role in addressing breakthrough pain, and being more explicit about it, was an important realization for our team, as controlling breakthrough pain is considered a key element of comprehensive cancer pain management [64]. In addition, we also emphasize that there are no “right or wrong” answers and added an “unsure” option to relevant EMA questions. A related issue was the temporal uncertainty of patients taking medication for a pain event. In other words, did they mark a pain event and *then* take pain medication, and if so, how much later? Or did they take pain medication and *then* mark a pain event afterward? We ultimately dealt with this thorny problem by revising our reassessment pain EMAs to retrospectively ask participants what was done to manage the pain and approximately what time the patient took their medication, if applicable.

We also found that participants, overall, accepted passive environmental monitoring and did not feel this compromised their privacy. However, it remains critical for researchers working in this field to be aware of, and sensitive to, concerns regarding environmental monitoring that may be particularly relevant for participant groups where long-standing systemic and structural factors have resulted in negative and discriminatory experiences related to such types of surveillance. Transparent informed consent, easy ways for participants to opt out (such as simply unplugging devices), and flexible monitoring protocols (eg, ones that can pivot to only active, user-initiated vs passive, environmental monitoring if needed or requested) are essential to ensure that systems such as BESI-C are culturally sensitive.

### Feasibility

#### Technical Feasibility: Fidelity of Data Capture; System Performance Scores

Our “BESI-C Performance Scoring Instrument” proved to be a helpful tool to assess holistic system functioning, while being able to identify trends regarding individual system components. To our knowledge, this is the first document created to monitor technology health modeled after clinical assessment tools.We suggest that this type of scoring sheet be adapted for other complex sensing systems or remote health monitoring systems to provide team members with a concise, clear, and quantifiable snapshot of system performance and a way to compare functioning and ensure a positive trajectory over time.

It is encouraging that the BESI-C overall composite performance scores increased over time, with a clear increase after our first deployment. Our scoring instrument confirmed that our environmental sensors had the most stable data-capture fidelity. This was not surprising, as this technology evolved from a previous, well-established project designed to monitor agitation in home-based patients with dementia and had more prior testing [65-67]. The primary concern regarding environmental sensors is aesthetics. Subsequent iterations resulted in a drastic reduction in size and a more streamlined design of our custom environmental relays without compromising the technical performance.

In contrast, the BESI-C smart watch app (the newest aspect of the system) proved to be less reliable, with inconsistent delivery of EMAs and challenges with battery life (our goal was 14 hours to increase the chance for 24 hours of continuous smart watch data, but we maxed out around 7 hours) and losing synchronization with the correct date and time. Unreliability of the smart watch app likely resulted in underreporting of pain events and contributed to other missing data. A key reason for these challenges with the smart watch app was automatic Android operating system updates, which affected system stability, a known challenge when using off-the-shelf commercial products [68]. On the basis of a review of our performance scores, after these 5 deployments, we migrated to a cloud services system to improve our ability to securely off-load and store data in real time.

A key technical lesson learned during these initial deployments was related to the importance of periodic code reviews and putting best practices in place regarding the software coding procedures. With each deployment, we learned new information regarding data capture that required iterative changes. However, the clinical team often underestimated the complexity or length of time needed to make, implement, and test these changes. Technical challenges reinforced the importance of clear, frequent, and transparent interdisciplinary communication as well as the importance of streamlining deployment procedures with this particularly sick and fragile patient population.

#### Procedural Feasibility: Deployment Processes; Participant Recruitment

Despite the known challenges of participant recruitment for palliative care–related research [53,55,69], we were able to successfully recruit 5 dyads (and expect this positive momentum would have continued if the COVID-19 pandemic had not interfered). Demographic trends must be interpreted cautiously given the sample size. However, we recruited patients with diverse cancer diagnoses, the majority with head and neck cancer, consistent with the high rates of tobacco use in our cancer center catchment area [70,71]. We also demonstrated the ability to recruit patients from groups at high risk of inadequate symptom management, including Black or African American and rural patients. This is important, as the most significant overarching goal of this research is to reduce cancer health disparities by increasing equitable access to quality cancer pain management.

Our study was complicated by the need for informed consent from both the patient and family caregiver. At times, this presented logistic challenges. For example, the patient’s family caregiver was not always physically present in the clinic when the study was discussed and the patient signed consent (this has become even more of a challenge with the COVID-19 pandemic and visitor restrictions). This resulted in one instance where the (consented) patient repeatedly assured the study team that he had discussed the study with his caregiver, who agreed to participate and would sign the informed consent form at home. However, when we arrived at the dyad home, our team quickly ascertained that the patient had not discussed the study with his wife. After careful discussion and emphasizing voluntary participation, the caregiver agreed, consented, and the deployment proceeded smoothly. After this experience, we made significant changes to our consenting procedures to ensure that if the caregiver is not with the patient at the time of the clinic visit, the caregiver is contacted before deployment, and interest in participating is directly confirmed by a study team member. We also learned the importance of deploying BESI-C as soon as possible after obtaining informed consent. Reducing time delays between consent and deployment proved essential to mitigate attrition and accommodate the dynamic clinical status of patients who are seriously ill.

Another primary recruitment challenge included screening potentially eligible clinic patients, as some key study criteria were not easily verifiable within the electronic health records. For example, it was difficult to determine whether the patient had a full-time family caregiver. We found that the most accurate (but not necessarily most efficient) way to identify potentially eligible patients was to discuss the daily clinic list face-to-face with the patient’s primary palliative care provider, who was more familiar with the nuances of the patient’s social context and clinical trajectory. Ultimately, we met our prestudy identified goal of 80% of enrolled dyads completing the full deployment (4 out of 5 completed the full deployment). We also set a prestudy goal of 50% of eligible dyads to enroll, but this proved difficult to accurately assess and reinforce the importance of having a stronger infrastructure in place for tracking participant screening, eligibility, enrollment, and reasons for not enrolling, such as with a REDCap (Research Electronic Data Capture; Vanderbilt University) database and a dedicated clinical research coordinator who could be physically present in clinic full-time to discuss the study with all eligible and interested participants.

**Figure 4:**
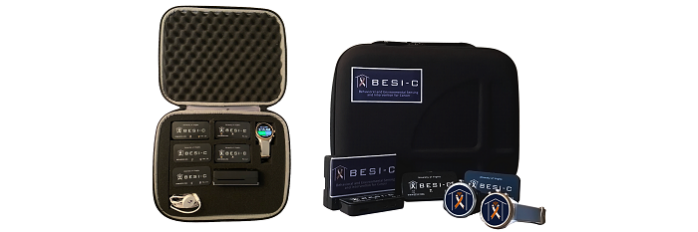
The “BESI Box” to facilitate “contactless” deployments.

Recruitment was also severely disrupted by the COVID-19 pandemic, which put a temporary halt on all human subject research and had a particularly negative impact on our research, which involved small research teams entering participant homes. We initially intended to recruit 15 dyads but were only able to complete 5 deployments before the COVID-19 restrictions were enacted. During this hiatus, we pivoted and completely redesigned our system to be contactless and allow for self-installation. This was a significant undertaking, from both the clinical and engineering sides of the project, but has resulted in a more scalable, streamlined system architecture (the “BESI Box” [72]) for future deployments (Figure 4). The “BESI Box” allows us to ship or drop off the system at participant homes and they can set it up themselves with remote support as needed.

With each deployment, our team became better and faster at setting up and removing the BESI-C system in participant homes. We also learned important lessons regarding the inherent challenges of in-home research. Specifically, we recognized the importance of explicit protocols for identifying and promptly responding to unexpected safety issues at home. For example, during a deployment, it was discovered that the participant home had multiple unsecured firearms whose locations interfered with sensor placement. This was detected by the engineering team members during the installation of environmental sensors in bedrooms and other living spaces but not by the nurse team members who remained in the living room teaching the caregiver about the smart watch app. Consequently, this critical information was not shared with the entire team until the return car ride. On the basis of this experience the team decided on an illogical but nonthreatening “safety phrase” (eg, “the server is down”) that would alert team members a huddle was immediately needed to reassess safety in the home.

### Limitations

The primary limitation of this study is the sample size, which reduces generalizability and the ability to detect statistical significance in our analysis of survey responses. However, our sample size is consistent with the scope of feasibility and acceptability studies that deploy complex remote health monitoring technology with actual patients [58,67,73] and addresses an important gap in reducing cancer health disparities in rural populations. It is also important to interpret our sample size in the context of the COVID-19 global pandemic, which completely halted participant recruitment during the second half of the funding period. We also had a sample of particularly dedicated and altruistic participants (screened and referred by palliative care staff) committed to making a broader scientific contribution. In addition, patients and caregivers answered postdeployment surveys and structured interview questions individually, but verbally in the presence of each other (deployments 1-4). Deployment 5 participants recorded their responses on separate iPads, which likely reduced potential response bias. Finally, as this was a feasibility and acceptability study (and not an efficacy or intervention trial), it was not our goal to use the collected data to directly help or modify patient or caregiver pain or distress; however, this is a key goal for future work*.*

### Conclusions

The BESI-C smart health remote monitoring system offers a holistic and innovative approach for monitoring and managing cancer pain in the home context. In this study, we successfully demonstrated the feasibility and acceptability of BESI-C using a sample of primarily rural patients with advanced cancer and their family caregivers. We also demonstrated the exciting possibilities of using heterogenous environmental, physiological, and behavioral sensing data to increase awareness and understanding of the cancer pain experience and promote enhanced communication among patients, caregivers, and health care providers. Future work will test the BESI-C in a larger and more diverse sample; continue to streamline system architecture; deploy a no-contact, self-installation system in response to the COVID-19 pandemic and to enhance scalability; explore how to best share data visualizations of collected data with key stakeholders; and design and deliver just-in-time personalized pain management interventions to patients and caregivers.

## Appendix

Multimedia Appendix 1 Details of the Behavioral and Environmental Sensing and Intervention for Cancer Scoring Instrument.

## Abbreviations

BESI-C: Behavioral and Environmental Sensing and Intervention for Cancer
ECOG: Eastern Cooperative Oncology Group
EMA: ecological momentary assessment
PROMIS: Patient-Reported Outcomes Measurement Information System
REDCap: Research Electronic Data Capture
