# Supplementary material for: Deploying the Behavioral and Environmental Sensing and Intervention for Cancer Smart Health System to Support Patients and Family Caregivers in Managing Pain: Feasibility and Acceptability Study
Source: JMIR Cancer. 2022 Aug 9;8(3):e36879. doi: 10.2196/36879 (PMC9399893; doi:10.2196/36879)
Supplement: Multimedia Appendix 1 [file cancer_v8i3e36879_app1.docx]

**Supplementary Data File

Details of Calculating the Performance Metrics**

In the wearable sensor category, we calculated “percent daily End-Of-Day (EOD) EMAs generated versus expected” simply by dividing the number of EOD EMAs generated on the wearable smart watch by the number of total deployment days. For example, for a 10-day deployment we would expect 10 EOD EMA surveys to be generated, 1 each evening. So, if over 10 days only 5 EOD EMAs were generated by the wearable, then the percent would be 50%, which is a score of 2. The metric “percent daily EOD surveys completed” refers to the number of EOD surveys that the user completed (i.e., answered all questions and submitted the EMA) divided by the number of EOD surveys generated on the wearable device. For example, if 5 EOD EMAs were generated and the participant completed all of them, then this would be 100%, and receive a score of 4. The metrics “percent follow-up EMAs generated versus expected” and “percent follow-up EMAs completed” were calculated in a similar fashion, accounting for our EMA schema which only generated follow-up EMAs if participants said they took pain medication.

‘Percent time watch worn’ was calculated using heartrate sensor data points which provided a proxy for the time the watch was worn on a participant’s wrist (i.e., if no heartrate data was detected and the watch was not on the charger, we considered that time as ‘watch not being worn.’) We calculated the metric ‘percent time watch worn’ by using the hours that we were able to track reliable heart rate data from the participant divided by the total hours in the deployment. For example, if 150 hours of heart rate data were collected from the patient over the course of a 10-day deployment (total 240 hours), then the percent time watch worn during deployment would be 62.5%, and this would receive a score of 3.

Battery life across the deployment was calculated by using a combination of: 1) average time between the wearable being off the charger and receipt of low battery notification; and 2) average time of wearable functioning between being charging sessions. For example, if on day 1 the battery lasted 9 hours (went from 100% to 0% in 9 hours), on day 2 the battery lasted 7 hours; and on days 3 – 10, the battery never went all the way to 0% because participant charged the watch before it died, then the average battery life across the deployment would be 8 hours, receiving a score of 2.

In the scoring category of environmental sensors, the metric “percent environmental data collected/expected” was calculated by pooling the respective sensing data from all sensors in the home and averaging the data collection minutes and then dividing by the number of total minutes in the deployment. For example, if light data were collected for 12,000 minutes in the bedroom, 10,500 minutes in the kitchen, and 13,000 minutes in the living room, this would equal an average light data collection of 11,833 minutes. This was then divided by the total number of minutes in a 10-day deployment (14,400), to equal 82.2%, receiving a score of 4.

Finally, the “percent of pain events at home with environmental data” refers to the number of times a pain event (either initial or follow-up) was recorded by a participant with available/collected environmental data within five minutes of the recorded pain event.

**BESI-C Performance Scoring Instrument Results, Per Deployment

Deployment 1**

| **Overall Deployment Days** | | | | | |  |
| --- | --- | --- | --- | --- | --- | --- |
|  | **0** | **7** | **14** | **21** | **28** | **SCORE** |
| Actual days of active data collection | <2 days | 2-3 days | 4-6 days | 7-9 days | ≥10 days | 28 |
| Total Score | | | | | | 28/28 |
| **Watch - Patient** | | | | | |  |
| Percent daily EOD EMAs *generated (expected)* | <10% | 11-30% | 31-50% | 51-80% | 81-100% | 0 |
| Percent daily EOD survey completed | <10% | 11-30% | 31-50% | 51-80% | 81-100% | 2 |
| Percent follow-up EMAs *generated (expected)* | <10% | 11-30% | 31-50% | 51-80% | 81-100% | 4 |
| Percent follow-up EMAs *completed (actual)* | <10% | 11-30% | 31-50% | 51-80% | 81-100% | 4 |
| Percent time watch worn over course of deployment (*calculated from HR data & when on charger*) | <10% | 11-30% | 31-50% | 51-80% | 81-100% | 3 |
| Average duration of battery life across deployment/hours per charge cycle | 1-3 | 3-6 | 6-9 | 9-12 | > 12 | 2 |
| Total Score | | | | | | 15/24 |
| **Watch – Caregiver** | | | | | |  |
| Percent daily EOD EMAs *generated (expected)* | <10% | 11-30% | 31-50% | 51-80% | 81-100% | 0 |
| Percent daily EOD survey completed | <10% | 11-30% | 31-50% | 51-80% | 81-100% | 2 |
| Percent follow-up EMAs *generated (expected)* | <10% | 11-30% | 31-50% | 51-80% | 81-100% | 3 |
| Percent follow-up EMAs *completed (actual)* | <10% | 11-30% | 31-50% | 51-80% | 81-100% | 2 |
| Percent time watch worn over course of deployment (*calculated from HR data & when on charger*) | <10% | 11-30% | 31-50% | 51-80% | 81-100% | 1 |
| Average duration of battery life across deployment/hours per charge cycle | 1-3 | 3-6 | 6-9 | 9-12 | > 12 | 2 |
| Total Score | | | | | | 10/24 |
| **Rooms (2 relays per room /minimum)** | | | | | |  |
| Percent data collected/expected for audio | <10% | 11-30% | 31-50% | 51-80% | 81-100% | 4 |
| Percent data collected/expected for light | <10% | 11-30% | 31-50% | 51-80% | 81-100% | 4 |
| Percent data collected/expected – weather (temp, pressure, humidity) | <10% | 11-30% | 31-50% | 51-80% | 81-100% | 4 |
| % of pain events (initial & follow up) at home w/ audio data | <10% | 11-30% | 31-50% | 51-80% | 81-100% | 4 |
| % of pain events (initial & follow up) at home w/ environmental data | <10% | 11-30% | 31-50% | 51-80% | 81-100% | 4 |
| % of pain events (initial & follow up) at home w/ light data | <10% | 11-30% | 31-50% | 51-80% | 81-100% | 4 |
| Total Score | | | | | | 24/24 |
|  |  |  |  |  |  |  |
| Total Deployment Score | | | | | | 77/100 |

**Deployment 2**

| **Overall Deployment Days** | | | | | |  |
| --- | --- | --- | --- | --- | --- | --- |
|  | **0** | **7** | **14** | **21** | **28** | **SCORE** |
| Actual days of active data collection | <2 days | 2-3 days | 4-6 days | 7-9 days | ≥10 days | 21 |
| Total Score | | | | | | 21/28 |
| **Watch - Patient** | | | | | |  |
| Percent daily EOD EMAs *generated (expected)* | <10% | 11-30% | 31-50% | 51-80% | 81-100% | 4 |
| Percent daily EOD survey completed | <10% | 11-30% | 31-50% | 51-80% | 81-100% | 4 |
| Percent follow-up EMAs *generated (expected)* | <10% | 11-30% | 31-50% | 51-80% | 81-100% | 4 |
| Percent follow-up EMAs *completed (actual)* | <10% | 11-30% | 31-50% | 51-80% | 81-100% | 3 |
| Percent time watch worn over course of deployment (*calculated from HR data & when on charger*) | <10% | 11-30% | 31-50% | 51-80% | 81-100% | 3 |
| Average duration of battery life across deployment/hours per charge cycle | 1-3 | 3-6 | 6-9 | 9-12 | > 12 | 3 |
| Total Score | | | | | | 21/24 |
| **Watch – Caregiver** | | | | | |  |
| Percent daily EOD EMAs *generated (expected)* | <10% | 11-30% | 31-50% | 51-80% | 81-100% | 4 |
| Percent daily EOD survey completed | <10% | 11-30% | 31-50% | 51-80% | 81-100% | 4 |
| Percent follow-up EMAs *generated (expected)* | <10% | 11-30% | 31-50% | 51-80% | 81-100% | 4 |
| Percent follow-up EMAs *completed (actual)* | <10% | 11-30% | 31-50% | 51-80% | 81-100% | 3 |
| Percent time watch worn over course of deployment (*calculated from HR data & when on charger*) | <10% | 11-30% | 31-50% | 51-80% | 81-100% | 3 |
| Average duration of battery life across deployment/hours per charge cycle | 1-3 | 3-6 | 6-9 | 9-12 | > 12 | 2 |
| Total Score | | | | | | 20/24 |
| **Rooms (2 relays per room /minimum)** | | | | | |  |
| Percent data collected/expected for audio | <10% | 11-30% | 31-50% | 51-80% | 81-100% | 4 |
| Percent data collected/expected for light | <10% | 11-30% | 31-50% | 51-80% | 81-100% | 4 |
| Percent data collected/expected – weather (temp, pressure, humidity) | <10% | 11-30% | 31-50% | 51-80% | 81-100% | 4 |
| % of pain events (initial & follow up) at home w/ audio data | <10% | 11-30% | 31-50% | 51-80% | 81-100% | 4 |
| % of pain events (initial & follow up) at home w/ environmental data | <10% | 11-30% | 31-50% | 51-80% | 81-100% | 4 |
| % of pain events (initial & follow up) at home w/ light data | <10% | 11-30% | 31-50% | 51-80% | 81-100% | 4 |
| Total Score | | | | | | 24/24 |
|  | | | | | |  |
| Total Deployment Score | | | | | | 89/100 |

**Deployment 3**

| **Overall Deployment Days** | | | | | |  |
| --- | --- | --- | --- | --- | --- | --- |
|  | **0** | **7** | **14** | **21** | **28** | **SCORE** |
| Actual days of active data collection | <2 days | 2-3 days | 4-6 days | 7-9 days | ≥10 days | 28 |
| Total Score | | | | | | 28/28 |
| **Watch - Patient** | | | | | |  |
| Percent daily EOD EMAs *generated (expected)* | <10% | 11-30% | 31-50% | 51-80% | 81-100% | 4 |
| Percent daily EOD survey completed | <10% | 11-30% | 31-50% | 51-80% | 81-100% | 4 |
| Percent follow-up EMAs *generated (expected)* | <10% | 11-30% | 31-50% | 51-80% | 81-100% | 4 |
| Percent follow-up EMAs *completed (actual)* | <10% | 11-30% | 31-50% | 51-80% | 81-100% | 3 |
| Percent time watch worn over course of deployment (*calculated from HR data & when on charger*) | <10% | 11-30% | 31-50% | 51-80% | 81-100% | 3 |
| Average duration of battery life across deployment/hours per charge cycle | 1-3 | 3-6 | 6-9 | 9-12 | > 12 | 2 |
| Total Score | | | | | | 20/24 |
| **Watch – Caregiver** | | | | | |  |
| Percent daily EOD EMAs *generated (expected)* | <10% | 11-30% | 31-50% | 51-80% | 81-100% | 3 |
| Percent daily EOD survey completed | <10% | 11-30% | 31-50% | 51-80% | 81-100% | 4 |
| Percent follow-up EMAs *generated (expected)* | <10% | 11-30% | 31-50% | 51-80% | 81-100% | 4 |
| Percent follow-up EMAs *completed (actual)* | <10% | 11-30% | 31-50% | 51-80% | 81-100% | 2 |
| Percent time watch worn over course of deployment (*calculated from HR data & when on charger*) | <10% | 11-30% | 31-50% | 51-80% | 81-100% | 2 |
| Average duration of battery life across deployment/hours per charge cycle | 1-3 | 3-6 | 6-9 | 9-12 | > 12 | 2 |
| Total Score | | | | | | 17/24 |
| **Rooms (2 relays per room /minimum)** | | | | | |  |
| Percent data collected/expected for audio | <10% | 11-30% | 31-50% | 51-80% | 81-100% | 4 |
| Percent data collected/expected for light | <10% | 11-30% | 31-50% | 51-80% | 81-100% | 4 |
| Percent data collected/expected – weather (temp, pressure, humidity) | <10% | 11-30% | 31-50% | 51-80% | 81-100% | 4 |
| % of pain events (initial & follow up) at home w/ audio data | <10% | 11-30% | 31-50% | 51-80% | 81-100% | 4 |
| % of pain events (initial & follow up) at home w/ environmental data | <10% | 11-30% | 31-50% | 51-80% | 81-100% | 4 |
| % of pain events (initial & follow up) at home w/ light data | <10% | 11-30% | 31-50% | 51-80% | 81-100% | 4 |
| Total Score | | | | | | 24/24 |
|  | | | | | | |
| Total Score | | | | | | 89/100 |

**Deployment 4**

| **Overall Deployment Days** | | | | | |  |
| --- | --- | --- | --- | --- | --- | --- |
|  | **0** | **7** | **14** | **21** | **28** | **SCORE** |
| Actual days of active data collection | <2 days | 2-3 days | 4-6 days | 7-9 days | ≥10 days | 28 |
| Total Score | | | | | | 28/28 |
| **Watch - Patient** | | | | | |  |
| Percent daily EOD EMAs *generated (expected)* | <10% | 11-30% | 31-50% | 51-80% | 81-100% | 4 |
| Percent daily EOD survey completed | <10% | 11-30% | 31-50% | 51-80% | 81-100% | 4 |
| Percent follow-up EMAs *generated (expected)* | <10% | 11-30% | 31-50% | 51-80% | 81-100% | 4 |
| Percent follow-up EMAs *completed (actual)* | <10% | 11-30% | 31-50% | 51-80% | 81-100% | 3 |
| Percent time watch worn over course of deployment (*calculated from HR data & when on charger*) | <10% | 11-30% | 31-50% | 51-80% | 81-100% | 3 |
| Average duration of battery life across deployment/hours per charge cycle | 1-3 | 3-6 | 6-9 | 9-12 | > 12 | 1 |
| Total Score | | | | | | 19/24 |
| **Watch – Caregiver** | | | | | |  |
| Percent daily EOD EMAs *generated (expected)* | <10% | 11-30% | 31-50% | 51-80% | 81-100% | 4 |
| Percent daily EOD survey completed | <10% | 11-30% | 31-50% | 51-80% | 81-100% | 2 |
| Percent follow-up EMAs *generated (expected)* | <10% | 11-30% | 31-50% | 51-80% | 81-100% | 3 |
| Percent follow-up EMAs *completed (actual)* | <10% | 11-30% | 31-50% | 51-80% | 81-100% | 3 |
| Percent time watch worn over course of deployment (*calculated from HR data & when on charger*) | <10% | 11-30% | 31-50% | 51-80% | 81-100% | 3 |
| Average duration of battery life across deployment/hours per charge cycle | 1-3 | 3-6 | 6-9 | 9-12 | > 12 | 3 |
| Total Score | | | | | | 18/24 |
| **Rooms (2 relays per room /minimum)** | | | | | |  |
| Percent data collected/expected for audio | <10% | 11-30% | 31-50% | 51-80% | 81-100% | 4 |
| Percent data collected/expected for light | <10% | 11-30% | 31-50% | 51-80% | 81-100% | 4 |
| Percent data collected/expected – weather (temp, pressure, humidity) | <10% | 11-30% | 31-50% | 51-80% | 81-100% | 4 |
| % of pain events (initial & follow up) at home w/ audio data | <10% | 11-30% | 31-50% | 51-80% | 81-100% | 4 |
| % of pain events (initial & follow up) at home w/ environmental data | <10% | 11-30% | 31-50% | 51-80% | 81-100% | 4 |
| % of pain events (initial & follow up) at home w/ light data | <10% | 11-30% | 31-50% | 51-80% | 81-100% | 4 |
| Total Score | | | | | | 24/24 |
|  |  |  |  |  |  |  |
| Total Deployment Score | | | | | | 89/100 |

**Deployment 5**

| **Overall Deployment Days** | | | | | |  |
| --- | --- | --- | --- | --- | --- | --- |
|  | **0** | **7** | **14** | **21** | **28** | **SCORE** |
| Actual days of active data collection | <2 days | 2-3 days | 4-6 days | 7-9 days | ≥10 days | 28 |
| Total Score | | | | | | 28/28 |
| **Watch - Patient** | | | | | |  |
| Percent daily EOD EMAs *generated (expected)* | <10% | 11-30% | 31-50% | 51-80% | 81-100% | 4 |
| Percent daily EOD survey completed | <10% | 11-30% | 31-50% | 51-80% | 81-100% | 4 |
| Percent follow-up EMAs *generated (expected)* | <10% | 11-30% | 31-50% | 51-80% | 81-100% | 1 |
| Percent follow-up EMAs *completed (actual)* | <10% | 11-30% | 31-50% | 51-80% | 81-100% | 3 |
| Percent time watch worn over course of deployment (*calculated from HR data & when on charger*) | <10% | 11-30% | 31-50% | 51-80% | 81-100% | 3 |
| Average duration of battery life across deployment/hours per charge cycle | 1-3 | 3-6 | 6-9 | 9-12 | > 12 | 4 |
| Total Score | | | | | | 19/24 |
| **Watch – Caregiver** | | | | | |  |
| Percent daily EOD EMAs *generated (expected)* | <10% | 11-30% | 31-50% | 51-80% | 81-100% | 3 |
| Percent daily EOD survey completed | <10% | 11-30% | 31-50% | 51-80% | 81-100% | 4 |
| Percent follow-up EMAs *generated (expected)* | <10% | 11-30% | 31-50% | 51-80% | 81-100% | 1 |
| Percent follow-up EMAs *completed (actual)* | <10% | 11-30% | 31-50% | 51-80% | 81-100% | 2 |
| Percent time watch worn over course of deployment (*calculated from HR data & when on charger*) | <10% | 11-30% | 31-50% | 51-80% | 81-100% | 3 |
| Average duration of battery life across deployment/hours per charge cycle | 1-3 | 3-6 | 6-9 | 9-12 | > 12 | 4 |
| Total Score | | | | | | 17/24 |
| **Rooms (2 relays per room /minimum)** | | | | | |  |
| Percent data collected/expected for audio | <10% | 11-30% | 31-50% | 51-80% | 81-100% | 4 |
| Percent data collected/expected for light | <10% | 11-30% | 31-50% | 51-80% | 81-100% | 4 |
| Percent data collected/expected – weather (temp, pressure, humidity) | <10% | 11-30% | 31-50% | 51-80% | 81-100% | 4 |
| % of pain events (initial & follow up) at home w/ audio data | <10% | 11-30% | 31-50% | 51-80% | 81-100% | 4 |
| % of pain events (initial & follow up) at home w/ environmental data | <10% | 11-30% | 31-50% | 51-80% | 81-100% | 4 |
| % of pain events (initial & follow up) at home w/ light data | <10% | 11-30% | 31-50% | 51-80% | 81-100% | 4 |
| Total Score | | | | | | 24/24 |
|  |  |  |  |  |  |  |
| Total Deployment Score | | | | | | 88/100 |
